# Lockjaw from a metastatic uterine leiomyosarcoma- case report and review of the literature

**DOI:** 10.1186/s12905-017-0472-1

**Published:** 2017-11-28

**Authors:** Isabel Hope, Karen Morton, Carrie Newlands, Simon Butler-Manuel, Thumuluru Kavitha Madhuri

**Affiliations:** 1 Faculty of Health & Medical Sciences, University of Southampton, Southampton, UK; 2 Royal Surrey County Hospital NHS Foundation Trust, Guildford, UK; 3 Department of Clinical & Experimental Medicine, Faculty of Health and Medical Sciences, University of Surrey, Guildford, UK

**Keywords:** Leiomyosarcoma, Metastases, Lockjaw

## Abstract

**Background:**

Leiomyosarcoma (LMS) is a malignant tumour formed of cells with distinct smooth muscle features. Leiomyosarcomas rarely metastasise to the oral cavity and this literature review details all reported cases of metastasis to the mandible found in the literature. This offers a unique perspective by specifying mandible as the site of metastasis of leiomyosarcoma.

**Case presentation:**

A 53-year-old female presented to her General Practitioner (GP) with heavy menstrual bleeding and was diagnosed with multiple fibroids. Folowing a hysterectomy and removal of both tubes and ovaries for these symptomatic uterine fibroids, an incidental diagnosis of low grade leiomyosarcoma was made. A CT scan found no evidence of residual or metastatic disease and no further treatment was deemed necessary. 6 months later she presented to A & E with a numb lower lip but it took another 6 months for the diagnosis of metastatic LMS to the mandible to be made.

**Discussion:**

Leiomyosarcomas are aggressive tumours which are liable to metastasise and therefore have a poor prognosis. An extensive literature review was undertaken to explore the frequency of metastasis in the maxillo-facial region.

**Conclusions:**

Although metastasis to the oral region is very rare as suggested from the literature review, when patients present with unusual symptoms after a diagnosis of LMS, physicians should be aware of the possibility of LMS metastases.

## Background

Leiomyosarcomas (LMS) are smooth muscle tumours arising from the embryonic mesenchyme cell lines. Uterine LMS are aggressive tumours often associated with a poor prognosis however a rare entity accounting for 1% of cases with a uterine malignancy. Treatment is surgical in the early stage when the disease is confined to the uterus. Recurrent disease is diagnosed by the development of new symptoms such as the index case. Metastases to the oral cavity are rare and we report an interesting case of uterine LMS with metastases to the mandible and review the literature to assess frequency of mandibular metastases.

## Case presentation

A 53 year old female presented to her General Practitioner with heavy menstrual bleeding and was referred to a gynaecologist at her local hospital. Her previous medical history included one normal vaginal delivery aged 28, a Mirena IUS in situ, and, a normal cervical screening history. Drugs prescribed included ranitidine and citalopram. Family history included an identified BRCA mutation carried by her mother and aunt. She had not been genetically tested herself but underwent regular 6 monthly checks from ages 41 to 50.

Multiple fibroids were identified on a trans-vaginal ultrasound of the pelvis. In view of her ongoing symptoms, the patient was offered a hysterectomy with a bilateral salpingo-oopherectomy.

The histology of the uterine specimen demonstrated one fibroid measuring 70 mm diameter and showing an abnormal focal area and central cystic necrosis consistent with a low grade leiomyosarcoma surrounded by normal tissue with no evidence of vascular infiltration. The leiomyosarcoma border was 10 mm clear of the serosal surface. A CT scan of the chest, abdomen and pelvis found no evidence of residual or metastatic disease and her case was discussed at a Gynaecologic Oncology multi disciplinary meeting (MDT). It was agreed that the likelihood of further metastasis was remote and she was discharged from follow-up.

Six months later, the patient presented to the accident and emergency department with a complaint of numbness in her lower left lip. A full neurological examination was normal so the patient was assured it would likely resolve. However, following a consultation with her dentist, she was referred to the maxillofacial department. The patient was known to grind her teeth due to anxiety. Altered sensation over left chin was reported, followed by numbness in her left lower lip. On examination, she was found to have decreased sensitivity to soft touch in the left mental nerve distribution area and an electric shock like sensation through her lip when pressure was applied to her mental foramen. Dental pulp testing with ethyl chloride was used on her lower left second premolar but no response was recorded and on X-ray, the apex was found to be directly next to the mental foramen and an apical radiolucency was associated with this tooth. She was treated with a course of amoxicillin on the diagnosis of an apical infection affecting the mental nerve. Despite the antibiotic treatment, the numbness continued and the patient went on to develop dysesthesia of the left 4th and 5th fingers. She also experienced an episode of severe left-sided facial pain associated with difficulty in opening her jaw; the left side of her chin became swollen and rubbery to touch. Her left lower second molar was treated with a root canal filling, which also failed to improve her symptoms. Further neurological examination confirmed trigeminal motor function was unaffected and Tinel’s sign was negative in the left elbow; peripheral nerve and Electromyography (EMG) studies were also found to be normal.

Magnetic resonance imagery of the brain and mandible along the course of the mandibular branch of the trigeminal nerve showed no evidence of metastasis. CT of the skull base, mandible and neck showed no bony abnormality and did not identify any prominent lymphadenopathy. Other investigations were undertaken, including chest X-ray, full blood count, ESR 5, clotting screen, B12 levels, plasma electrolytes, calcium, C reactive protein, angiotensin converting enzyme levels, liver function tests, thyroid function and blood sugar levels which were all normal. Tumour markers were tested and normal as well as Wassermann reaction, anti-nuclear factor, anticardiolipin antibodies, ANCA (antineutrophil cytoplasmic antibodies) screen and borrelia titres were negative, making any infective or autoimmune causes less likely. The patient declined to have a lumbar puncture (LP), instead wishing to try a course of acupuncture first. After completing her sessions of acupuncture over 4 weeks, she felt that the sensory disturbance in her left hand and left chin had improved by about 40%. She therefore decided to carry on with another four sessions of acupuncture before considering a LP. Having finished the acupuncture, the patient found her left hand sensory disturbance to be almost completely resolved and her left chin was continuing to improve. She was prescribed antibiotics by her dentist to treat an abscess of the lower left wisdom tooth however this did not resolve the problem and led to a swelling in her left cheek. The left lower third molar was removed, and while extracting the tooth, a biopsy of abnormal ‘moth eaten’ looking soft tissue was also taken.

**Fig. 1:**
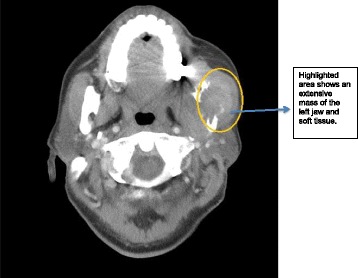
showing a contrast enhanced axial CT of the neck. Highlighted area shows an extensive mass of the left jaw and soft tissue

**Fig. 2:**
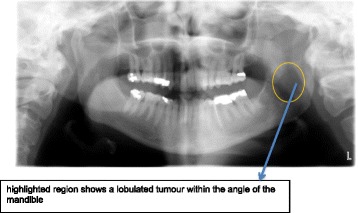
Orthopantogram (OPG), highlighted region shows a lobulated tumour within the angle of the mandible

The histopathology report of the biopsy revealed an undifferentiated sarcomatous malignant tumour. Immunohistochemistry staining was used to compare this specimen to the uterine LMS from the hysterectomy previously and was consistent with recurrent disease. A second CT of the neck, abdomen and pelvis performed after an interval of 6 months revealed an extensive mass lesion affecting the left jaw and soft tissue mass on both sides (Fig. 1). A dental panoramic radiograph also showed a lobulated tumour within the angle of the mandible (Fig. 2). The final diagnosis of metastatic uterine LMS to the left mandible and masseter was made, over 6 months after the patient had initially presented with jaw paresthesia.

Primary treatment was resection of the masseter, medial pterygoid and angle of the mandible. A positron emission tomography (PET) scan was suggestive of small metabolically active area in pelvis, possibly further nodal spread, but it was not macroscopically visible on CT so no further excision was recommended.

Radiotherapy to the left mandibular lesion followed, with clinical follow-up. Further metastases subsequently developed with two lesions in the lungs, and one in the left pelvic side-wall. Diagnosis was made after a PET scan and the pelvic mass was treated with resection and radiotherapy. A pathological fracture to the humerus, secondary to the bony LMS metastasis at the site, lead to surgical correction and further radiotherapy. She later developed a metastatic lesion within the duodenum and underwent a pancreatic duodenectomy with resection of metastasis. An osteolytic mass was then identified in the right 4th rib and local palliation radiotherapy was administered. After further disease progression, 6 cycles of chemotherapy and palliative radiotherapy to the right iliac area, she died of progressive disease, 4 years following the original diagnosis of uterine LMS.

### Literature review

A review of the literature was undertaken to assess frequency of mandibular metastases from uterine LMS. Databases including MEDLINE, EMBASE and google scholar were searched, the search being limited to the English language but with no time limit. Searching of the grey literature was also undertaken. Articles containing case reports of LMS metastasising to the ‘oral cavity’ were then further analysed to find cases specifically involving the mandible as a site of metastasis. From this thorough search, LMS metastasizing to the mandible was only reported in seven other cases, three from the uterus [1–3] as in this case, three from the lower limb [4, 5] and one from the abdomen (pancreas, abdominal aorta and vena cava) [6] (Table 1).

**Table 1:** Results from literature search. Leiomyosarcomas metastasising to the mandible. Year journal article was published, author of journal article, primary site of leiomyosarcoma, metastatic sites, prognosis/follow up since metastatic diagnosis

Year	Authors	Age/Sex	Primary Site	Metastatic Site	Survival
1986	Tsounias et al.	67/F	Uterus	Mandible, lung, bones	2 months died
1993	Allen et al.	61/M	Thigh	Mandible, liver, spleen, lung, kidney	3 years follow up
2000	Dry SM et al.	45/F	Uterus	Maxilla, mandible	11 months died
2011	Azevedo et al.	69/M	Pancreas, abdominal aorta, vena cava	Liver, mandible	1 year died
2011	Jham BC et al.	60/M	Leg	Mandible	
2011	Jham BC et al.	69/M	Leg	Mandible	
2013	Fernandez-Barriales et al.	49/F	Uterus	Mandible, masseter, maxilla, lung, liver, vertebral body, lymph node	18 months died
2015	Hope et al.	54/F	Uterus	Mandible, left masseter, humerus, duodenum	4 years died

The index case is only the 4th reported case of uterine LMS metastasizing to the mandible found by this extensive search of the literature (Table 2) and highlights how rare the oral region is as a metastatic site, particularly as a first site of recurrent disease.

**Table 2:** All cases of uterine LMS metastasising to the mandible found in literature

Year	Author	Age	Metastatic Sites	Treatment of Uterine Primary	Treatment of Metastases	Disease free interval	Survival from diagnosis	Survival after appearance of metastasis	Cause of death	First symptoms of metastasis	Method of diagnosis
1988	Tsounias et al.	67	Mandible, lung, bones	S	S (curettage)	12 months (uterine then mandible)	1 year 2 months	2 months	Metastatic disease	Pain in left mandible	X-ray then surgery
2000	Dry SM et al.	45	Maxilla, mandible	None	S + R	1 month (uterine after mandible)	11 months	11 months	Metastatic disease	Loose teeth + left nasal congestion	Unknown
2013	Fernandez-Barriales et al.	49	Mandible, masseter, maxilla, lung, liver, vertebral body, lymph node	C + R	C + R	0 months (simultaneous uterine + mandible)	1 year 6 months	18 months	Metastatic disease	Hypoesthesia in inferior alveolar nerve territory (NCS)	Observation of mass then OPG
2015	Hope et al.	54	Mandible, left masseter, humerus, duodenum	S	S + R + C	14 months (uterine then mandible)	4 years 2 months	3 years	Progressive metastatic disease	Left lower lip hypoesthesia (NCS)	Tooth extraction, biopsy of tissue

Year of article publication, author, age of patient at diagnosis, metastatic sites of the uterine LMS, treatment of primary tumour and subsequent metastases, survival time from diagnosis, survival time after metastatic diagnosis, cause of death, first reported symptom of metastatic spread, method of metastatic diagnosis.

## Discussion

Leiomyosarcoma (LMS) is a malignant tumour formed of cells with distinct smooth muscle features [7]. Uterine LMS originates from myometrium and represents 40% of uterine sarcomas [8] but with an incidence of only 0.4–0.64/100,000 women [8, 9]. 131,467 people were diagnosed with cancer in the UK in 2011. Of these 403 were women diagnosed with uterine sarcoma, 161 of these being uterine LMS.

Patients presenting with uterine LMS have a median age of 55 years and commonly present with vaginal bleeding (56%), a palpable pelvic mass (54%) and pelvic pain (22%) [10]. However, most uterine leiomyosarcomas are detected during the histopathological analysis of a hysterectomy or myomectomy specimen [11] probably because these presenting symptoms are very similar with benign leiomyomas [12]. Uterine LMS most commonly spreads hematogenously to intra-abdominal viscera, lung, pleura para-aortic nodes and kidneys [10]. The prognosis in uterine LMS is poor in all stages of the disease and appears to be dependent on menopausal state. If the diagnosis is made in pre-menopausal women, the 5-year survival rate is 63.6% whereas in post-menopausal women the 5-year survival rate drops to 5.5% [10, 13]. This may be due to the hormonal environment, because preservation of the ovaries also seems to improve prognosis [11].

Pathologically, LMS structure is irregular and large with necrosis, haemorrhage, atypical nuclei and often with extra uterine extension [14]. Magnetic resonance imaging (MRI) will show a large composite mass that warps uterine architecture and contains necrosis and haemorrhage.

This case is unusual due to the location of the metastasis, as the oral cavity and oropharynx are rare sites of metastases from primary cancers of any organ. In fact, only 1–2% of oral malignant tumours are metastases from other primary sites [15]. However, the mandible is the most common site of these metastases to the oral cavity, with one paper [16] reporting that 55% of metastases to the oral cavity are to the mandible.

Clinicians need to remain suspicious of metastases in any site following treatment for a primary LMS. LMS are difficult to diagnose initially and there may be an inevitable delay in diagnosis due to investigation into infectious or erosive causes. However if a patient has a previous history of LMS, a metastasis should always be considered if they present with unusual symptoms, including in the jaw as in our patient. Consideration of metastatic LMS in a patient with a previous diagnosis is likely to allow for a swift diagnosis, and a possible improved prognosis. In this index case, it took over 6 months after the patient presented with sensory defects to be diagnosed with a metastasis to the jaw. Delay in the diagnosis and therefore treatment of metastatic LMS has the potential to allow the cancer to develop and increase the chance of further spread. A delay in diagnosis also prevents the onset of palliative care and leaves the patient symptomatic.

As can be seen by Table 2, this index case had the longest survival of all cases seen in the literature, at 4 years from presentation. Survival is influenced by many factors, the most influential in LMS being volume of tumour [6], presence of coagulative tumour cell necrosis, mitotic figures and cytologic atypia [17]. However, the aggressive treatment given in this index case could have played a part in her prolonged survival, with each new recurrence being treated with either surgery, chemotherapy, radiotherapy or a combination of the three. This contrasts with Tsounias et al.’s [1] treatment of solely surgery, and Fernandez-Barriales et al.’s [3] administration of chemotherapy and radiotherapy for the simultaneously diagnosed uterine primary and mandibular metastasis.

The referral of the index case to the supraregional centre for sarcomas may have improved outcome, due to experienced staff optimising the treatment given to this patient. At the time of referral, their diagnostic resources were superior to those in the local hospital: for example they could use PET to detect further metastases that were not apparent on CT.

## Conclusions

This case demonstrates the possibility of LMS metastasising to the jaw and affecting nerves in this region, proving the aggressive nature of the tumour. If there is a recent history of leiomyosarcoma and a patient presents with nonspecific or unusual symptoms or with sensory disturbance in any system of the body, a secondary leiomyosarcoma should be considered in the differential diagnosis. Aggressive treatment in a specialist centre combined with the continued identification of new metastases may improve survival.

## Abbreviations

A & E: Accident & Emergency
ANCA: Antineutrophil cytoplasmic antibodies
BRCA: Breast Cancer susceptibility gene
CT scan: Computed Tomography scan
EMG: Electromyography
GP: General Practitioner
IUS: Intra Uterine System
LMS: Leiomyosarcoma
LP: Lumbar puncture
MDT: Multidisciplinary Team meeting
MRI: Magnetic resonance imaging
PET: Positron emission tomography
